# Supplementary material for: Trifluoperazine and Its Analog Suppressed the Tumorigenicity of Non-Small Cell Lung Cancer Cell; Applicability of Antipsychotic Drugs to Lung Cancer Treatment
Source: Biomedicines. 2022 Apr 30;10(5):1046. doi: 10.3390/biomedicines10051046 (PMC9138877; doi:10.3390/biomedicines10051046)
Supplement: Supplementary file 1 [file biomedicines-10-01046-s001.zip › biomedicines-1670748-supplementary.pdf]

Figure S1

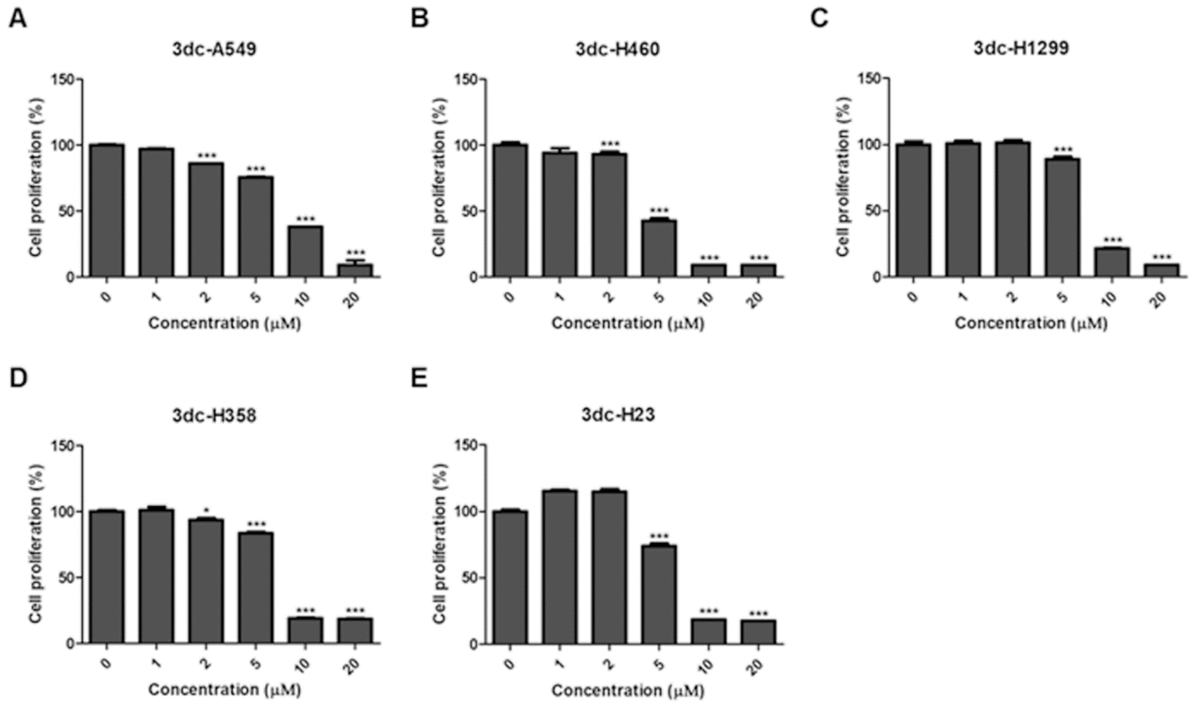

Effect of 3dc on NSCLC cell proliferation. NSCLC cells (A549, H460, H1299, H358, H23) were treated with 3dc at various concentrations (0-20 μM). Cell proliferation was assessed using MTT assay. Data shown as mean ± S.E. $*P < 0.05$, $***P < 0.001$ *vs*. CTL (0 μM for each).
